# The Actin Targeting Compound Chondramide Inhibits Breast Cancer Metastasis via Reduction of Cellular Contractility

**DOI:** 10.1371/journal.pone.0112542

**Published:** 2014-11-12

**Authors:** Magdalena H. Menhofer, Rebekka Kubisch, Laura Schreiner, Matthias Zorn, Florian Foerster, Rolf Mueller, Joachim O. Raedler, Ernst Wagner, Angelika M. Vollmar, Stefan Zahler

**Affiliations:** 1 Department of Pharmacy, Pharmaceutical Biology, University of Munich, Munich, Germany; 2 Department of Pharmacy, Pharmaceutical Biotechnology, University of Munich, Munich, Germany; 3 Department of Physics, Soft Matter Physics and Biophysics, University of Munich, Munich, Germany; 4 Helmholtz Institute for Pharmaceutical Research Saarland, Helmholtz Centre for Infection Research and Department of Pharmaceutical Biotechnology, Saarland University, Saarbrücken, Germany; The Beatson Institute for Cancer Research, United Kingdom

## Abstract

**Background:**

A major player in the process of metastasis is the actin cytoskeleton as it forms key structures in both invasion mechanisms, mesenchymal and amoeboid migration. We tested the actin binding compound Chondramide as potential anti-metastatic agent.

**Methods:**

*In vivo*, the effect of Chondramide on metastasis was tested employing a 4T1-Luc BALB/c mouse model. *In vitro*, Chondramide was tested using the highly invasive cancer cell line MDA-MB-231 in Boyden-chamber assays, fluorescent stainings, Western blot and Pull down assays. Finally, the contractility of MDA-MB-231 cells was monitored in 3D environment and analyzed via PIV analysis.

**Results:**

*In vivo*, Chondramide treatment inhibits metastasis to the lung and the migration and invasion of MDA-MB-231 cells is reduced by Chondramide *in vitro*. On the signaling level, RhoA activity is decreased by Chondramide accompanied by reduced MLC-2 and the stretch induced guanine nucleotide exchange factor Vav2 activation. At same conditions, EGF-receptor autophosphorylation, Akt and Erk as well as Rac1 are not affected. Finally, Chondramide treatment disrupted the actin cytoskeleton and decreased the ability of cells for contraction.

**Conclusions:**

Chondramide inhibits cellular contractility and thus represents a potential inhibitor of tumor cell invasion.

## Introduction

Metastasis is the primary cause of death in cancer [1]. One reason for this is a lack of efficient therapies against metastasis. Currently, metastatic tumors can only be treated once they have established. Therapies targeting the initial steps of metastasis (local invasion, intravasation, arrest and extravasation) are still lacking, but might be a clinically attractive option, e.g. in cases where tumor resection might cause spreading of malignant cells. The formation of cancer colonies at sites distant from the primary tumor requires cellular properties distinct from those within the primary tumor. Thus, established therapies against primary tumors like doxorubicin or cisplatin are less effective against invasive cells. The resistance of invasive cells arises from the upregulation of anti-apoptotic genes and the downregulation of pro-apoptotic genes compared to primary tumor cells [2]. In addition to this anti-apoptotic phenotype, invasive cells show a far more plastic and migratory phenotype. This phenotype is characterized by cellular deformation involving the formation of protrusions and new adhesions to surfaces as well as cellular contractility, which is required for rear retraction and cellular transmigration [3]. In all these processes, the actin cytoskeleton plays a pivotal role and underlies a constant re- and disassembly to form protrusions and stress fibers [4], [5]. The importance of the actin cytoskeleton during metastasis is reflected at the level of actin regulating proteins as many of those are deregulated in metastatic cells [6], [7].

Invasive cells show two modes of migration: first, the mesenchymal type and, second, the amoeboid type of migration [8]–[10], which both depend on contractility. In the mesenchymal migration, cells show an elongated morphology with lamellipodial protrusions and high proteolytic activity, which is required in addition to contractility. Contrarily, the amoeboid migration mode is totally independent of proteolysis and can be induced as an escape mode when proteolysis is inhibited. In this migration type, cells show rounded shape and invasion through the matrix is primarily mediated via cellular contractility. The underlying signaling pathway involves Rho/ROCK and myosin to induce the actin dependent contractile force. In both processes, the actin cytoskeleton plays a central role either for the formation of protrusions or the contraction of the cell. Therefore, the actin machinery represents an attractive target to address metastatic cells.

Interestingly, a large number of natural compounds bind the actin cytoskeleton and interfere with actin dynamics [11]. Since actin is of central importance for most organisms, including parasites and fungi, nature most likely has favored the evolution of secondary metabolites which target actin one way or the other. Nevertheless, the use of these natural compounds has been neglected due to the ubiquitous occurrence of actin and possible severe side effects. However, the successful clinical use of microtubule binding agents as anti-cancer drugs mitigates this argument [12]. An actin binding compound evolved from natural selection is Chondramide. Chondramide is a cyclodepsipeptide isolated from the myxobacterium Chondromyces crocatus [13]. It induces actin polymerization *in vitro* and has only been shown to be cytotoxic but its detailed mode of action on cancer cells has not been elucidated so far [14].

We hypothesize that metastasis can be inhibited using the actin polymerizing compound Chondramide and elucidate its potency *in vitro* and *in vivo*. Here, we can show that Chondramide inhibits cancer cell migration and invasion *in vitro* involving signaling pathways, which influence cellular contractility. Further, we demonstrate that Chondramide is capable to reduce metastasis *in vivo*.

## Methods

### Material

Chondramide was isolated as previously described [15] and dissolved and stored in DMSO. For experiments, Chondramide (Ch) was dissolved in growth medium containing DMSO at a maximum of 0.1% (v/v).

### Cell culture

All cell lines were cultured under constant humidity at 37°C and with 5% CO2 in an incubator (Heraeus, Hanau, Germany). MDA-MB-231 cells were purchased from Cell Line Services (Eppelheim, Germany) and cultivated in dulbecco's modified eagle's medium (DMEM, PAN Biotech, Aidenbach, Germany) supplemented with 10% FCS. 4T1-Luc cells were purchased from Caliper Life Sciences (Alameda, CA, USA).

### *In vivo* experiment

20 BALB/c mice were divided into two groups (treatment and control) and inoculated via the tail vein with 1×10^5^ syngenic mammary tumor cells stably transfected with the luciferase gene (4T1-Luc cells). The treatment group was premedicated intravenously with 0.5 mg/kg Ch in 5% DMSO/PBS 24 h and 4 h before tumor cell injection. The control group was injected with equal amounts of 5% DMSO/PBS. On day eight after tumor cell inoculation, mice were anesthetized with 2% isoflurane in oxygen and 6 mg Na-luciferin were injected intraperitoneally. Thereafter, mice were sacrificed through cervical dislocation. Lungs were harvested and imaged using the IVIS Lumina system with Living Image software 3.2. Images were interpreted with equalized color bar scales, measuring the photon emission of the mice. For quantification of the lung signals, the images of the harvested lungs were analyzed. Regions of Interest (ROIs) were defined and total signals per ROI were calculated as photons/second/cm2 (total flux/area). During the experiment, mice were weighed every second till third day. All animal experiments were conducted according to the guidelines of the German law for protection of animal life and approved by the Government of Upper Bavaria (Permit No. 55′′′′.2-1-54-2532-107-13).

### Migration and invasion assays

For Boyden chamber assays, 5×10^4^ MDA-MB-231 or 4T1-Luc cells were seeded per well in a Boyden chamber with a pore size of 8 µM (Corning, New York, USA) without FCS. For negative control, the lower compartment was filled with medium lacking FCS, whereas for positive control and treated samples, the lower compartment was filled with full medium containing 10% FCS. After 16 h, cells were fixed and stained with crystal violet/methanol. Cells on top of the filter were removed with a q-tip and bottom sides were photographed using an Axiovert25 microscope (Zeiss), 10× objective, and a Canon EOS 450C camera (Tokyo, Japan). Images were analyzed using the ImageJ plugin cell counter. The invasion assay was performed analog to Boyden chamber assays except for filters being coated with 100 µl 10% matrigel (Schubert&Weiss-OMNILAB, Munich, Germany) and polymerized before seeding and an incubation time of 48 h.

### Fluorescence imaging

MDA-MB-231 cells were seeded in an IBIDI-μSlide (IBIDI, Martinsried, Germany) and cultivated overnight plus treatment period. F-actin was stained with rhodamin-phalloidin (1∶400, R 415, Molecular Pobes/Invitrogen) and nuclei with bisBenzimide H33342 (Sigma-Aldrich, St. Louis, MO, USA). The following antibodies were used: Integrin α5 (Millipore, Upstate), p(S19)-MLC (Cell Signalling Technology, Danvers, MA, USA) and Vinculin (Santa Cruz Biotechnology, Santa Cruz, CA, USA). Images were obtained with a Zeiss LSM 510 META (Zeiss, Oberkochen, Germany) or Leica TCS SP8 SMD confocal microscope (Leica, Mannheim, Germany).

### Adhesion assay

Pretreated MDA-MB-231 cells were trypsinized, suspended in DMSO or Ch containing medium and allowed to adhere on fibronectin, collagen G or plastic for 1 h. Cells were fixed with 4% para-formaldehyde and stained for F-actin. Images were taken on a Zeiss LSM 510 META confocal microscope (10× objective) and counted for adhering cells.

### Western blotting

Western blot analysis was performed as described previously [16]. The following antibodies were used: GAPDH, MLC2, Vav2, p(Y172)Vav2 (Santa Cruz Biotechnology), p(S473)Akt, Akt, p(Y1068)EGF-R, EGF-R, p(T202,Y204)Erk, Erk, p(S19)MLC2 (Cell Signalling Technology), Rac (23A8, Merck Millipore, Darmstadt, Germany), Rho (Thermo Scientific, Bonn, Germany).

### Rho GTPase pull down experiments

Activation of Rac1 and Rho were induced by adding 100 ng/ml EGF for 5 min to the medium followed by a pull down assay according to manufacturer's protocol (Thermo Scientific, Bonn, Germany).

### Contractility measurement

Yellow-green microbeads of 1 µm diameter, 2% solids (LifeTechnologies, Carlsbad, CA, USA) were mixed with matrigel in a ratio of 1∶4 (v/v) on ice. The bead/matrigel mixture was mixed with pretreated MDA-MB-231 cells, 5×106 cells/ml, in a ratio of 2∶1 (v/v) and pipetted into an angiogenesis slide (ibidi, Martinsried, Germany). Matrigel was allowed to polymerize for 1 h at 37°C, 5% CO2 in humidified atmosphere, before samples were covered with culture medium. Images were taken using an open U-iMIC fluorescent microscope (TILL Photonics GmbH, Gräfelfing, Germany) with 20× objective. Stacks of 5 slices around the cell equatorial plane with a 2 µm distance were taken over 4 h with 15 min intervals. Three optical slices at the equatorial plane were used for maximum intensity projection (imageJ) and images were standardized to 300×300 px with one cell at the image center. Movies of fluorescent beads were analyzed by particle image velocimetry (PIV) analysis with a customized MatPIV software package for Matlab. Interrogation windows were 32×32 pixels, i.e. 21×21 µm. A single iteration was performed with 62.5% overlap. The resulting velocity vectors were filtered with the set of filters included in the standard MatPIV package to smooth the vector fields. As the sample observation is restricted to 2D, z-components of the velocity are not seen and the PIV analysis only gives velocities projected into the observation plane. The obtained velocity field was averaged on each grid point over each frame of the observation period to further remove fluctuations that occur on short time scales. For quantitative comparison, the radial velocity towards the cell center per data was calculated. Minimum 13 cells per condition out of three independent experiments were recorded and analyzed.

### Measurement of sub-diploid DNA content

To detect apoptotic cells, sub-diploid DNA content (i.e. fragmented nuclei) was measured as described by Nicoletti et al. [17]. Cells were permeabilized and stained with propidiumiodide (50 µg/ml), followed by flow cytometry using a FACSCalibur (Becton Dickinson, Heidelberg, Germany).

### Statistical analysis

The number of independently performed experiments and the statistical tests used are stated in the respective figure legend. Graph data represent means ± SEM. Statistical analysis was performed with the software GraphPad Prism Version 5.04 (GraphPad Software, Inc., La Jolla, CA, USA). Statistical significance is assumed if p≤0.05. For the in vivo metastasis assay the statistical power was calculated to be 0.85 using G*power software.

## Results

### Chondramide diminishes metastasis *in vivo*

**Figure 1:**
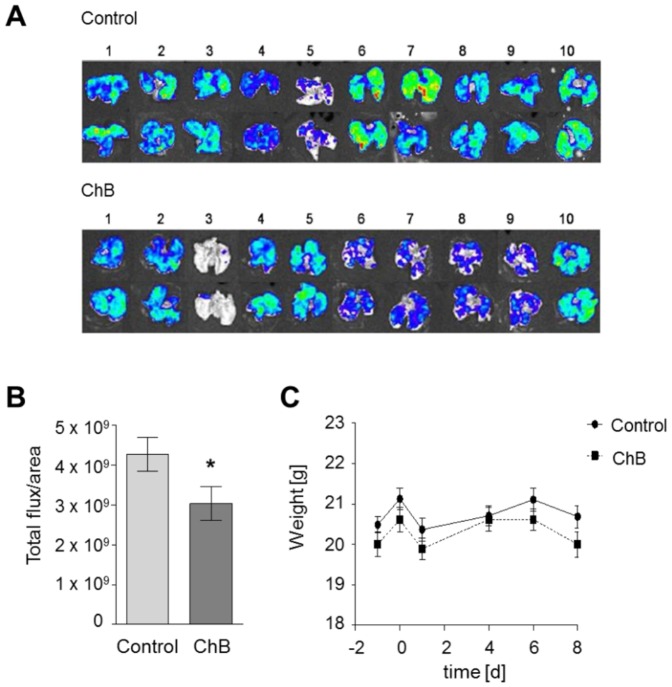
Chondramide diminishes metastasis *in vivo*. 1×10^5^ 4T1-Luc cells were injected intravenously into pretreated (0.5 mg/kg ChB) and untreated BALB/cByJRj mice. (A) 8 days after cell inoculation mice were sacrificed, lungs were harvested and used for recording bioluminescence signals. Each lung was imaged from the dorsal and ventral side. Color bar scales were equalized. (B) Quantitative evaluation of metastasis to the lungs. Region of interest were defined (ROI) and total luciferin signal in ROIs was calculated as photons/second/cm^2^ (total flux/area). Ten lungs per group, *, p<0.05 (t-test, unpaired). (C) Mouse weight over treatment period. Weight of treated (ChB) and untreated (DMSO) mice from day of cell inoculation (day 0) to euthanasia (day8) is shown for each group.

First, we evaluated the pharmacological, anti-metastatic potential of Chondramide (Ch) *in vivo*. To this end, we employed a 4T1-Luc metastatic mouse breast cancer model [18]–[20]. Hereby, a murine 4T1-Luc mammary cancer cell line expressing recombinant luciferase is injected intravenously in BALB/cByJRj mice. These luciferase expressing cancer cells metastasize to the lung and can be detected via bioluminescent measurement. The evaluation of the bioluminescent signal in the lungs of untreated and Ch treated mice revealed a significant reduction of metastasis for Ch treated mice compared to control mice (Fig. 1a,b). Over the treatment period, the weight of Ch treated mice stayed constant indicating a good tolerance of 0.5 mg/kg Ch during application (Fig. 1c).

### Chondramide affects stress fibers of highly invasive cancer cells

**Figure 2:**
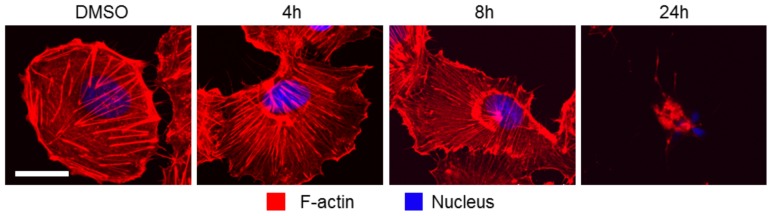
Chondramide leads to reduction of stress fibers and F-actin aggregation. MDA-MB-231 cells were treated with 200 nM ChA or DMSO for indicated time periods, fixed and stained for F-actin and nuclei (n = 3). Bar represents 20 µm.

As a model for highly invasive cancer cells the cell line MDA-MB-231 was used for *in vitro* experiments. In actin stainings of MDA-MB-231 cells, stress fibers seemed altered as compared to control cells. Since in parallel actin aggregates close to the nucleus after 4 and 8 h were observed (Fig. 2), this might be due to depletion of available actin. Alternatively, binding of the F-actin probe phalloidin might have been competitively inhibited by Chondramide. After 24 h the cells were completely condensed while no induction of apoptosis was observed (Fig. S1). These condensed cells recovered to the original cell size when Ch was removed (Fig. S2).

### Chondramide abrogates EGF induced migration, invasion and adhesion *in vitro*

**Figure 3:**
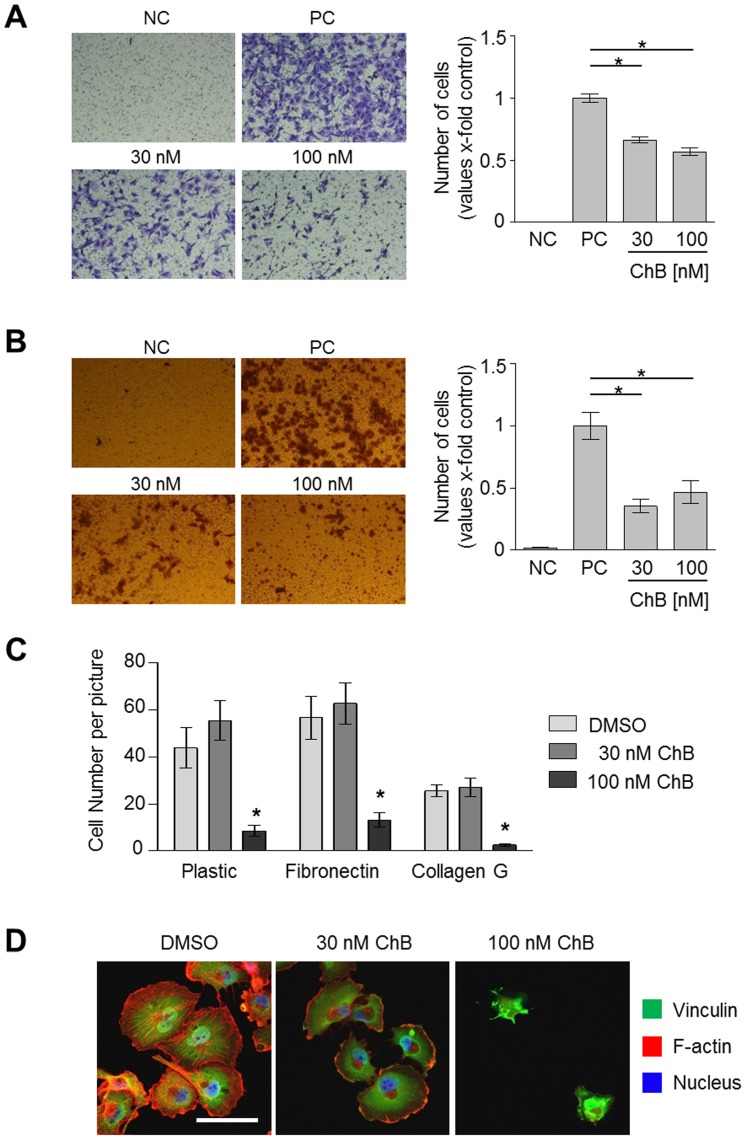
Treatment with Chondramide reduces breast cancer cell migration, invasion and adhesion. (A) Chondramide treated and untreated MDA-MB-231 cells were allowed to migrate in a Boyden chamber for 16 h. (B) Chondramide inhibits invasion of MDA-MB-231 cells through matrigel in Boyden chamber (48 h). A,B: For positive control (PC) lower compartment was filled with medium plus 10% FCS, for negative control (NC) only medium without FCS was added. *, p<0.05 One-way ANOVA, Tukey post-test, n = 3. (C) Pretreated MDA-MB-231 cells were seeded freshly on indicated surfaces and counted after fixation. *, p<0.05 One-way ANOVA, Tukey post-test, n = 3. (D) Cells from C were stained for F-actin, nuclei and vinculin. Bar represents 50 µm.

Next, the effect of Ch on the transmigration of MDA-MB-231 was tested in a Boyden chamber assay. The migration was inhibited significantly by Ch at 30 nM or 100 nM down to 70% or 60%, respectively (Fig. 3a). Similar effects (62% and 54%, respectively) were observed with the 4T1-Luc cells, which were also used for the in vivo assay (Figure S3). To study the effect of Ch on invasiveness, matrigel filled Boyden chambers were used. Here, Ch decreased cell invasion at 30 nM and 100 nM to less than 50% (Fig. 3b).

To further elucidate the effect of Ch on cell migration, a central step, cell adhesion, was tested. Here, Ch reduced the attachment of cells to plastic, fibronectin and collagen G at 100 nM, however, not at 30 nM (Fig. 3c). This indicates, that anti-adhesive properties of Ch could contribute to an anti-migratory effect, yet, this seems not to be the only factor diminishing cancer cell migration, at least at 30 nM concentration of the compound. Fluorescent stainings of the adhering cells revealed that cells treated with 30 nM Ch showed normal cell morphology whereas the few adhering cells at 100 nM Ch were not able to spread (Fig. 3d), which also indicated additional mechanisms at 30 nM Ch.

### Chondramide lowers the activity of the RhoGTPase Rho but not Rac1

**Figure 4:**
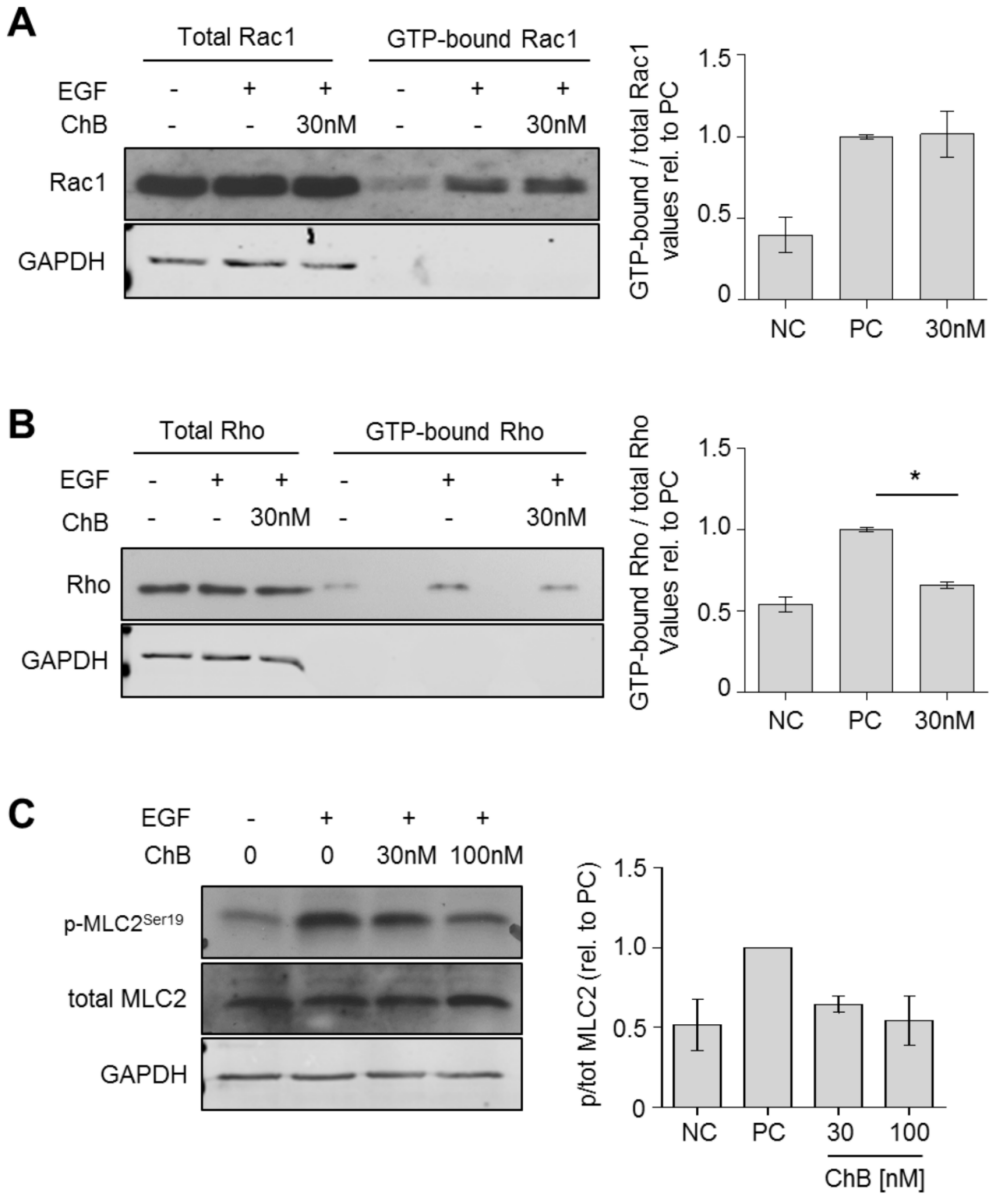
Chondramide affects activation of the RhoGTPase Rho. (A) A Rac1 pull down was performed for untreated and Chondramide treated cells (24 h) upon EGF-stimulation (5 min). (B) After same treatment a Rho pull down was conducted. (C) Myosin light chain 2 (MLC2) was analyzed on its activation state via Western blot analysis upon EGF stimulation (5 min). A,B,C: Left panel: one representative Western blot is shown. Right panel: Densitometric analysis of Western blots. *, p<0.05 One-way ANOVA, Tukey post-test, n = 3.

To investigate whether stress fiber reduction seen in Fig. 2b is a direct effect of Ch or induced due to alterations in signaling, the upstream effectors of the actin-cytoskeleton were tested for their activity. Therefore, the RhoGTPases Rac1 and Rho were analyzed using pull-down assay. We could see no alterations in the amount of GTP-bound Rac1 (Fig. 4a). However, following EGF stimulation, the level of GTP-bound Rho was clearly reduced at 30 nM Ch (Fig. 4b). The diminished activity of Rho could be confirmed further downstream as the regulatory myosin light chain 2 (MLC2) was less phosphorylated at Ser19 after treatment with Ch (Fig. 4c).

### Chondramide has no effect on the activation of the EGF-receptor and downstream signaling

**Figure 5:**
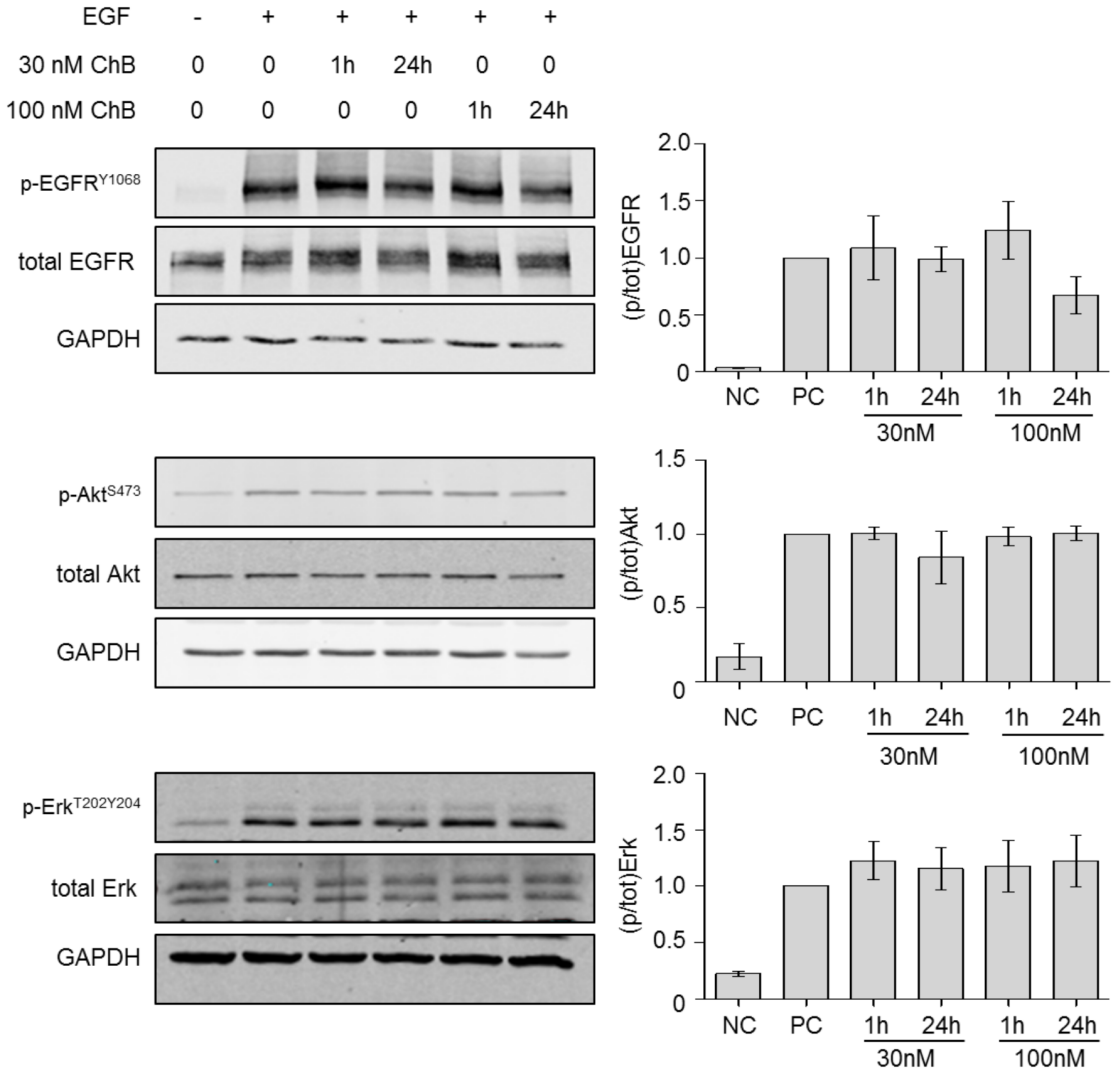
Chondramide does not influence EGF-R signalling. MDA-MB-231 cells were treated with Chondramide (1 or 24 h), stimulated with EGF (5 min) and analyzed via Western blot analysis on EGFR, Akt and Erk. Left panel: one representative Western blot is shown. Right: Densitometric analysis of Western blots. *, p<0.05 One-way ANOVA, Tukey post-test, n = 3.

Next, the underlying mechanism of reduced Rho activity by Ch was evaluated. As the Rho GTPases were activated through extracellular EGF stimulation in our experiments, we examined the EGF-receptor (EGFR) on its ability for autophosphorylation. The autophosphorylation at Y1068 of the EGFR was not affected by Ch treatment (Fig. 5). Neither 30 or 100 nM Ch nor a very short or longer time point showed any changes in the phosphorylation state. Concomitant with that, the phosphorylation status of the downstream effectors to EGFR, Akt and Erk, at S473 or T202Y204, respectively, showed no effect of Ch treatment compared to positive control (Fig. 5). This data indicates normal activation of these pathways under Ch treatment.

### Chondramide diminishes cellular contractility and the Rho activating GEF Vav2

**Figure 6:**
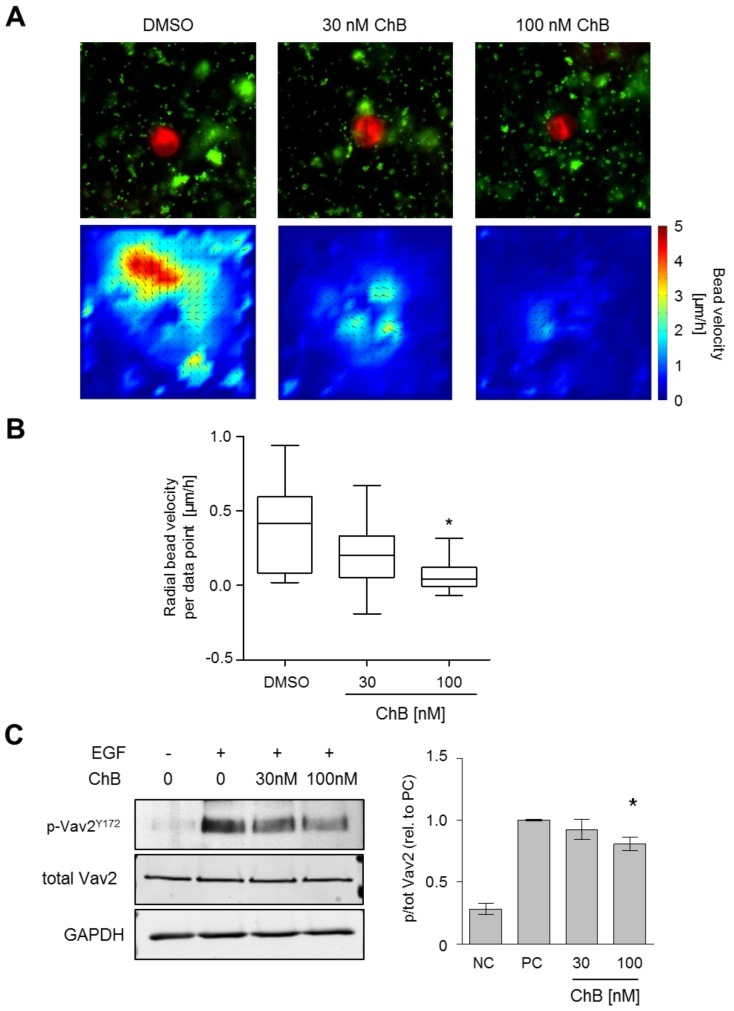
Chondramide diminishes contractility. (A) MDA-MB-231 cells were pretreated for 24 h as indicated, embedded in matrigel containing fluorescent beads and pictures were taken over 4 h every 15 min. Cellular, contractile force on the surrounding matrix was visualized via bead movement towards the cell and analyzed using PIV analysis. Upper panel: Representative images of cells (red) and fluorescent beads (green) t = 0. Lower panel: PIV analysis of bead velocity in the 2D projection. Color codes show bead velocity indicating applied force. Direction of vectors indicates averaged direction of bead movement. (B) For quantitative analysis the radial velocity (bead velocity towards the cell center) per data point was calculated. Minimum 13 cells per condition were analyzed. *, p<0.05 One-way ANOVA, Tukey post-test. (C) Phosphorylation of the Rho-GEF Vav2 was tested via Western blot analysis upon EGF stimulation (n = 3); * p<0.05 vs. control, One-way ANOVA, Tukey post-test.

The activation of the RhoGTPase Rho can also be mediated via intra- or extracellular forces. Thus, we tested the ability of MDA-MB-231 cells to exert contractile force. Cellular force on surrounding matrix can be visualized as exerted forces result in matrix deformation which can be imaged by embedded fluorescent beads [21], [22]. To this end, cells were seeded in matrigel containing fluorescent beads and images were taken over 4 h. Control cells revealed a clear contractile force on the surrounding matrix as seen by bead movement towards the cell (Fig. 6a, and movies S1 and S2). The bead movement was reduced at 30 nM and even more at 100 nM Ch (Fig. 6b), thus, indicating a reduction of intracellular originating force in cells treated with Ch.

Intra- and extracellular force lead to the activation of guanine nucleotide exchange factors (GEFs), e.g. Vav2, which activates RhoA. The activity dependent phosphorylation site Y172 of Vav2 was less phosphorylated under Ch treatment (Fig. 6c) indicating reduced activation of the GEF Vav2 after Ch application.

## Discussion

Cellular contractility is of utmost importance in cancer cell invasion. Classic migration depends on contractility as the cell rear has to be retracted after cell protrusion and formation of new adhesions in the migration direction. Further, the amoeboid migration of cancer cells is mostly driven by contractile force depending on Rho/ROCK signaling but not proteolysis [9], [10]. According to literature, the highly invasive cell line MDA-MB-231 follows the scheme of an amoeboid migration through matrigel, requiring a contractile uropod dependent on RhoA and MLC-2 activity [22]. During this process, cells exert contractile force to the surrounding matrix. Accordingly, we observed a matrix deformation by migrating MDA-MB cells, as shown by fluorescent bead movement. This bead displacement was reduced after Chondramide treatment indicating reduced contractile force of the cell due to treatment. The observation that migration, invasion and contractility are inhibited at the same concentration of Chondramide suggests that the reduction of contractility is the underlying cause for these functional defects.

The signaling cascade leading to contractility, via Rho/ROCK and myosin, is known to enhance invasion when upregulated [23], [24]. Chondramide treatment diminishes the activity of Rho and MLC2. Other factors like Rac1 or EGFR and its downstream factors, Akt and Erk, were not affected at same conditions. Thus, Chondramide primarily inhibits the pro-contractile signaling cascade.

Interestingly, a connection between contractile force and RhoA activation is reported not only from Rho being required for contractility but also the other way around that contractile force induces RhoA activation [25]. In this context, Vav2 has been shown to be induced by applied forces in the form of stretching in mesangial cells [26] and Vav2 is known to be necessary for a full activation of RhoA in MDA-MB-231 cells [27]. These observations are in line with our findings revealing that Vav2 and RhoA are less active in Chondramide treated cells. According to our in vitro data, adhesion is not yet affected at 30 nM Chondramide, while transmigration and Rho activity and phorphorylation of MLC2 are. We suggest that Chondramide may reduce contractility via reduced intra-cellular force diminishing Rho activation.

The approach to target the actin cytoskeleton has been thought to be too toxic for clinical application [28]. However, the feasibility to target actin is shown here in an *in vivo* mouse model of tumor metastasis. Herein, treatment with Chondramide diminishes the metastasis of mammary cancer cells to the lung significantly. As migration is reduced at lower concentrations and shorter time points than nuclear fragmentation occurs, we propose that the inhibitory effect on metastasis is not due to an apoptotic effect but the abrogation of migration and invasion. The dose of 0.5 mg/kg was well tolerated as the body weight of treated mice stayed constant throughout the observation period. Although there is still room for further development, e.g. specific targeting, we could show a pharmacological effect for Chondramide on metastasis *in vivo* without acute toxicity. In our in vivo treatment regime we tried to keep close to a potential clinical setting, and, thus, chose systemic administration of Chondramide. This, of course, means that cells other than tumor cells (e.g. endothelial cells) might have been affected by the compound, and that we cannot dissect, which step of metastasis might have been primarily affected (adhesion, extravasation, migration as such, trapping of tumor cells in the pulmonary microcirculation). Due to the short presence of compound (administration 24 h and 4 h before cell application vs. detection of metastasis after 8 days), it is unlikely that later steps of metastasis formation (survival, growth) might have been influenced by Chondramide.

As therapies against cancer metastasis are still missing, more efforts need to be made in research addressing metastatic cancer cells. This work provides evidence that actin is a suitable target to inhibit cancer cell migration, and gives first insights into underlying mechanisms. Combining actin binding natural compounds with specific targeting strategies could lead to a powerful weapon to treat tumor metastasis.

## Supporting Information

Figure S1 **Reduction of migration is not due to cell death.** Nuclear fragmentation was measured in MDA-MB-231 cells after ChA treatment and PI staining. *, p<0.05 One-way ANOVA, Tukey post-test, n = 3.
(TIF)

Figure S2 **Aggregation of cells is reversible.** MDA-MB-231 cells were treated with DMSO, 200 nM ChA for 40 h or with 200 nM ChA for 24 h and then additional 16 h without ChA. Cells were fixed and stained for F-actin and nuclei (n = 3). Scale bar represents 50 µm.
(TIF)

Figure S3 **Reduction of migration in 4T1-Luc cells.** Chondramide treated and untreated 4T1-Luc cells were allowed to migrate in a Boyden chamber for 16 h. For positive control (PC) lower compartment was filled with medium plus 10% FCS, for negative control (NC) only medium without FCS was added. *, p<0.05 One-way ANOVA, Tukey post-test, n = 3.
(TIF)

Movie S1 **Chondramide diminishes contractility.** MDA-MB-231 cells were untreated (movie 1) or pretreated for 24 h with 100 nM chondramide, embedded in matrigel containing fluorescent beads and pictures were taken over 4 h every 15 min. Red: labeled cell, green: fluorescent beads. Bead movement toward the cell (contractility) is much lower in the treated sample. For quantitative evaluation see Figure 6.
(AVI)

Movie S2 **Chondramide diminishes contractility.** MDA-MB-231 cells were untreated (movie 1) or pretreated for 24 h with 100 nM chondramide, embedded in matrigel containing fluorescent beads and pictures were taken over 4 h every 15 min. Red: labeled cell, green: fluorescent beads. Bead movement toward the cell (contractility) is much lower in the treated sample. For quantitative evaluation see Figure 6.
(AVI)
