# Supplementary figures and images for: The Actin Targeting Compound Chondramide Inhibits Breast Cancer Metastasis via Reduction of Cellular Contractility
Source: PLoS One. 2014 Nov 12;9(11):e112542. doi: 10.1371/journal.pone.0112542 (PMC4229209; doi:10.1371/journal.pone.0112542)

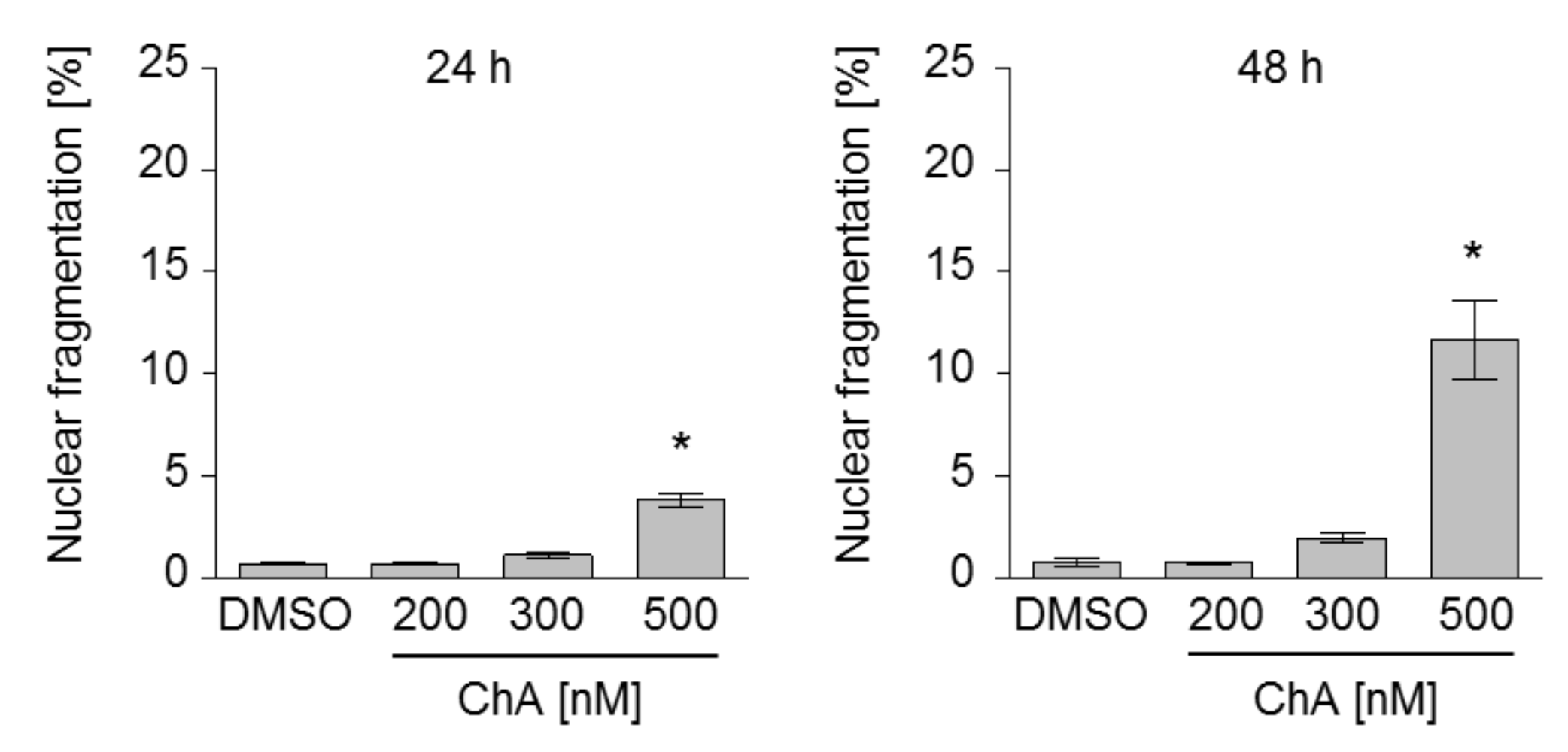

Supplement: Figure S1 — Reduction of migration is not due to cell death. Nuclear fragmentation was measured in MDA-MB-231 cells after ChA treatment and PI staining. *, p<0.05 One-way ANOVA, Tukey post-test, n = 3. (TIF) [file pone.0112542.s001.tif]

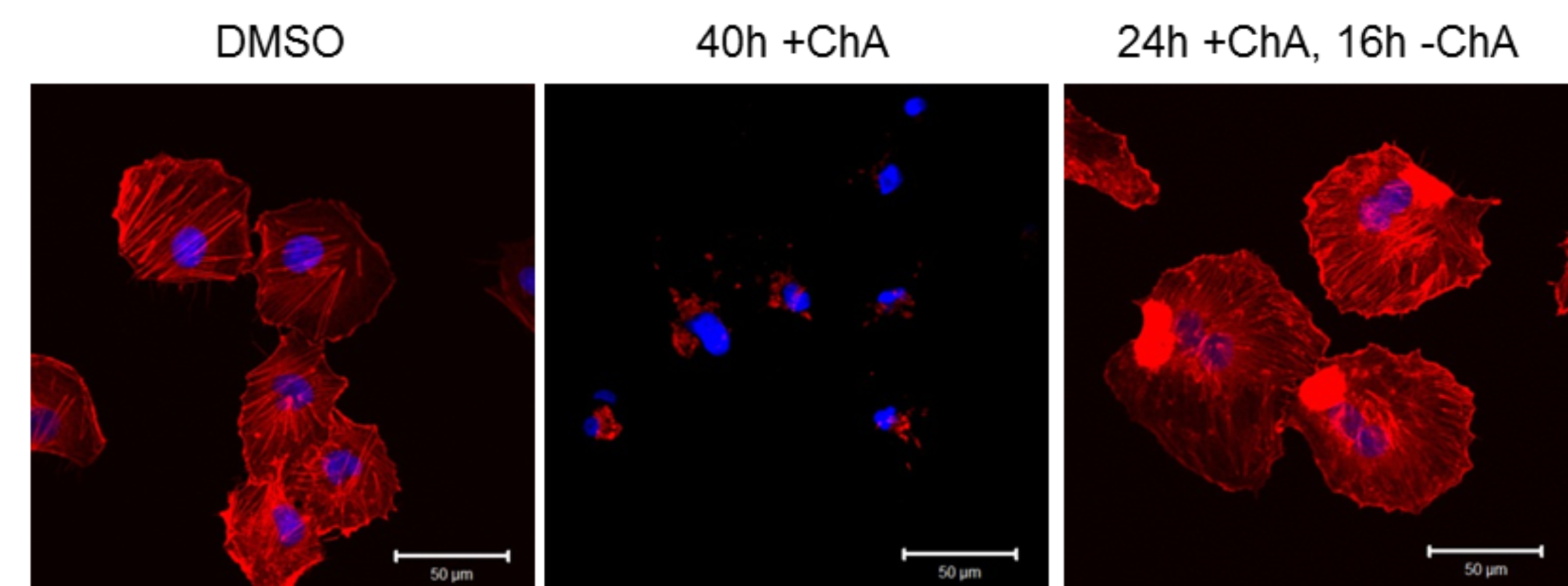

Supplement: Figure S2 — Aggregation of cells is reversible. MDA-MB-231 cells were treated with DMSO, 200 nM ChA for 40 h or with 200 nM ChA for 24 h and then additional 16 h without ChA. Cells were fixed and stained for F-actin and nuclei (n = 3). Scale bar represents 50 µm. (TIF) [file pone.0112542.s002.tif]

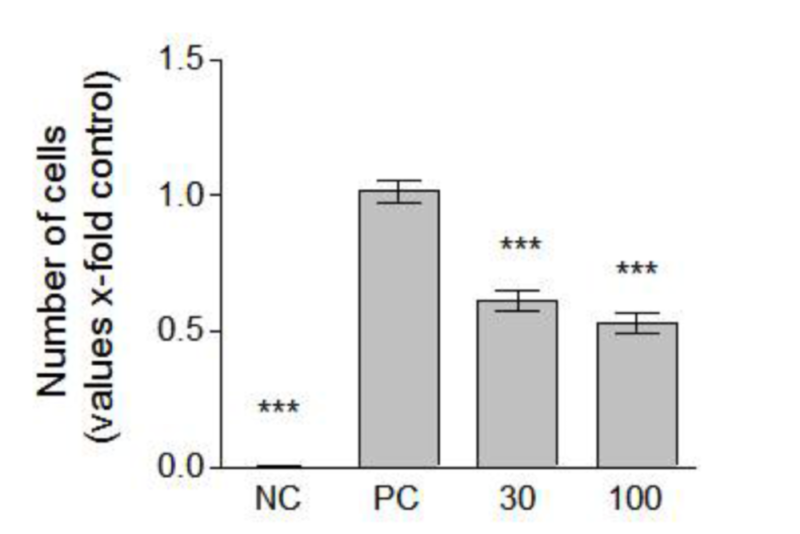

Supplement: Figure S3 — Reduction of migration in 4T1-Luc cells. Chondramide treated and untreated 4T1-Luc cells were allowed to migrate in a Boyden chamber for 16 h. For positive control (PC) lower compartment was filled with medium plus 10% FCS, for negative control (NC) only medium without FCS was added. *, p<0.05 One-way ANOVA, Tukey post-test, n = 3. (TIF) [file pone.0112542.s003.tif]
